# The impact of pharmaceutical form and simulated side effects in an open-label-placebo RCT for improving psychological distress in highly stressed students

**DOI:** 10.1038/s41598-023-32942-5

**Published:** 2023-04-19

**Authors:** Alexander Winkler, Alannah Hahn, Christiane Hermann

**Affiliations:** Department of Clinical Psychology and Psychotherapy, Justus-Liebig-University Giessen, Otto-Behaghel-Str. 10F, 35394 Giessen, Germany

**Keywords:** Placebo effect, Psychiatric disorders, Depression

## Abstract

Open-label placebo (OLP) may be utilized to reduce psychological distress. Yet, potential contextual effects have not been explored. We investigated the impact of pharmaceutical form and the simulation of side effects in a parallel group RCT (DRKS00030987). A sample of 177 highly stressed university students at risk of depression were randomly assigned by computer generated tables to a 1-week intervention with active or passive OLP nasal spray or passive OLP capsule or a no-treatment control group. After the intervention, groups differed significantly in depressive symptoms but not regarding other outcomes of psychological distress (stress, anxiety, sleep quality, somatization), well-being or treatment expectation. OLP groups benefitted significantly more compared to the no-treatment control group (*d* = .40), OLP nasal spray groups significantly more than the OLP capsule group (*d* = .40) and the active OLP group significantly more than the passive OLP groups (*d* = .42). Interestingly, before intervention, most participants, regardless of group assignment, believed that the OLP capsule would be most beneficial. The effectiveness of OLP treatments seems to be highly influenced by the symptom focus conveyed by the OLP rationale. Moreover, pharmaceutical form and simulation of side effects may modulate efficacy, while explicit treatment expectation seems to play a minor role.

## Introduction

For university students, elevated psychological distress (e.g., emotional suffering, stress, anxiety, depressed mood) has been reported, with prevalence rates up to 80%^1^. In order to cope with stress, a growing number of people resort to medication such as antidepressants^2^ or benzodiazepines^3^ as well as over-the-counter medications^4,5^, despite a lack of evidence for their effectiveness for stress reduction and their potential associated risks (e.g., abuse and unwanted side effects).

While there is robust experimental evidence^6–10^ that placebo treatments can positively affect emotional states and psychological well-being in healthy participants and even in depressive patients, placebos are still not used regularly in clinical and nonclinical contexts^11^. Presumably, hesitation to use placebos results from the belief that deception is necessary to induce placebo effects^12^, thus raising ethical and legal concerns^13^. However, meta-analytic findings^14^ suggest that placebo effects can be induced by honestly prescribed open-label placebos (OLPs), thus complying with the American Medical Association’s principles of medical ethics^15^. Indeed, in a first experimental OLP trial with healthy participants using sad-mood induction prior to and after OLP intervention, Hahn et al.^16^ demonstrated that active OLP nasal spray treatment, delivered with a convincing rationale for its sadness-protecting effect, effectively attenuated the experimentally induced increase in perceived sadness.

To date, there are only two intervention studies investigating OLP effects on stress, psychological distress and emotional well-being in students. Kleine-Borgmann et al.^17^ investigated a 21-day OLP intervention focusing especially on improving cognitive functioning in medical students during their exam preparation. While test performance was not improved, students taking the OLP pills reported less increase in perceived stress and less deterioration of subjective well-being and perceived stress. Hence, OLP may counteract negative effects of acute stress on psychological well-being. As the authors note, conclusions are limited due to external factors not being controlled for, such as other coping strategies during the exam preparation. Notably, the OLP intervention itself did not entail an elaborated rationale delivered face-to-face as in most other OLP studies. El Brihi et al.^18^ demonstrated substantial effects of OLP pills on psychological distress, positive mental well-being, physical symptoms and sleep quality in a 5-day OLP trial comparing different dosages. Due to the study’s reliance on the composite score of the Depression Anxiety Stress Scale 21 (DASS-21;^19^), specific OLP effects on depressive vs. anxiety symptoms or tension/stress cannot be disentangled. While OLP dose did not influence treatment outcome, treatment expectancies and treatment adherence were significant predictors of most well-being outcomes. Moreover, healthy students were included irrespective of their subjective stress level, thus limiting the generalizability of the findings for individuals with elevated distress levels.

Treating highly stressed students is not only relevant for assessing OLP interventions as a potential alternative to active medications for reducing psychological distress. Elevated psychological distress and stress is a risk factor for severe psychiatric disorders including major depression^20^. Indeed, prevalence rates of depression and other mental disorders are substantially higher in university students (mean prevalence: 30.6%) than those found in the general population^21^. In a study by Dickhäuser et al.^22^, perceived stress was significantly correlated with depressive symptoms (*r*=.71) in a sample of 1396 university students. Moreover, given that a large proportion (up to 70%) of the overall symptom improvement in antidepressant trials can be attributed to placebo effects^23^, utilizing honestly prescribed open-label placebos (OLPs) may constitute a promising and yet ethical way to help highly distressed students.

Currently, there are three OLP treatment studies in patients with major depressive disorder (MDD). In a feasibility study, Kelley et al.^24^ examined the effect of 14 days OLP in 20 patients with MDD. While clinicians rated the OLP patients as less depressed at the end of the treatment, there was no statistically significant difference compared to the waitlist group, presumably due to the low statistical power and short treatment duration. Nitzan et al.^25^ investigated 28 days OLP as an add-on to treatment-as-usual in 38 patients with depression. Depressive symptoms decreased significantly, regardless of the additional intake of OLP pills. Schienle et al.^26^ examined OLP oil as an adjunctive therapy in 60 patients with MDD participating in a 4-week cognitive behavioral therapy (CBT) outpatient program. The added OLP did not yield a clinically significant symptom reduction^26^. Patients were instructed that the daily oral intake of 3 drops of OLP oil could help with the relaxation exercises at home, hence the rationale did not specifically emphasize an impact on mood. Taken together, these studies suggest that it might be necessary to first learn more about the differential impact of contextual factors (e.g., treatment rationale, form of OLP) on the outcome of OLP interventions in nonclinical samples prior to making use of OLP in clinical populations.

Several mechanisms identified in experimental deceptive placebo research, such as physician–patient interaction, associative learning and treatment expectancy are presumed to at least partially account for the OLP effect as well^12,27–29^. Unconscious expectations induced by contextual factors and bodily sensations could also contribute to the OLP effect, taking into account that some participants report no or only minor explicit expectancy for improvement before treatment^28,30^. OLP treatment might activate pharmacological memories by the mere intake of some medication-like form (i.e., the placebo pill or capsule) and its sensory features serve as a conditioned cue that elicits previously experienced symptom improvement^12^. This assumption is supported by experimental studies demonstrating that the conditioned placebo response persisted even after the placebo manipulation was revealed to the participants^31,32^. According to Kaptchuk^28^ and Ballou et al.^33^, the assumption of unconscious processes being involved in the OLP effect is supported by theories like predictive processing and embodied cognition^34–36^, and neuroimaging evidence on the conditioning of placebo responses with nonconscious stimuli^37–39^. According to the concept of embodied cognition, the physical interaction with the environment affects our cognitions and vice versa^36,40^. For instance, during OLP treatment the same sensory and motor information is transmitted to the brain as during an active medical treatment, since it is experienced in a professional “medical” context signaling symptom improvement^28,33^. According to predictive coding, the brain corrects the mismatch between the sensory input associated, for example, with the intake of a capsule in a medical environment and the prediction that a placebo will not lead to a symptom improvement in favor of the sensory input, supporting symptom improvement via the release of neurotransmitters in the brain^12,33^. In light of these theoretical considerations, more salient sensory inputs (e.g., invasive interventions) should provoke stronger OLP effects independent of conscious treatment expectation. Indeed, there is empirical evidence for the influence of physical characteristics (e.g., larger pills, stronger taste, higher price) and the invasiveness of a treatment on its effectiveness^41,42^. For instance, invasive interventions (e.g., acupuncture, injections, surgery) induce greater health benefits than treatments that are less salient and intrusive, such as orally taken medications^41,43^. Similarly, active placebos (i.e., placebos combined with a pharmacological substance that induces noticeable sensations (“side effects”, but without effect on the target symptom) can induce stronger placebo effects than inert placebos^44^. Whether this is also true for OLP treatments has not yet been investigated. Clearly, this would have direct implications on how to design optimal clinical OLP interventions.

The aim of this study was to determine to what extent the effectiveness of an OLP intervention for reducing psychological distress might depend on the pharmaceutical form of the OLP and simulated side effects. Specifically, an OLP nasal spray and an OLP capsule were compared, which differ not only with regard to their sensory features, but possibly also with regard to their familiarity depending on the number of previous treatment experiences and the type and range of symptoms having been targeted by such forms of medication. Moreover, an active vs. a passive nasal spray were compared, thus allowing the role of distinct sensations, presumably indicating the onset of the treatment effect, to be delineated. Using an analogue sample approach, and based on previous reports of high correlations between stress and depressive symptoms in students^22^, the OLP treatments were evaluated in highly stressed university students with elevated depressive symptoms. We expected a more pronounced reduction of psychological distress in the OLP groups compared to the natural history group, in the OLP nasal spray groups compared to the OLP capsule group and in the active OLP group compared to the passive OLP groups.

## Method

### Participants

**Figure 1:**
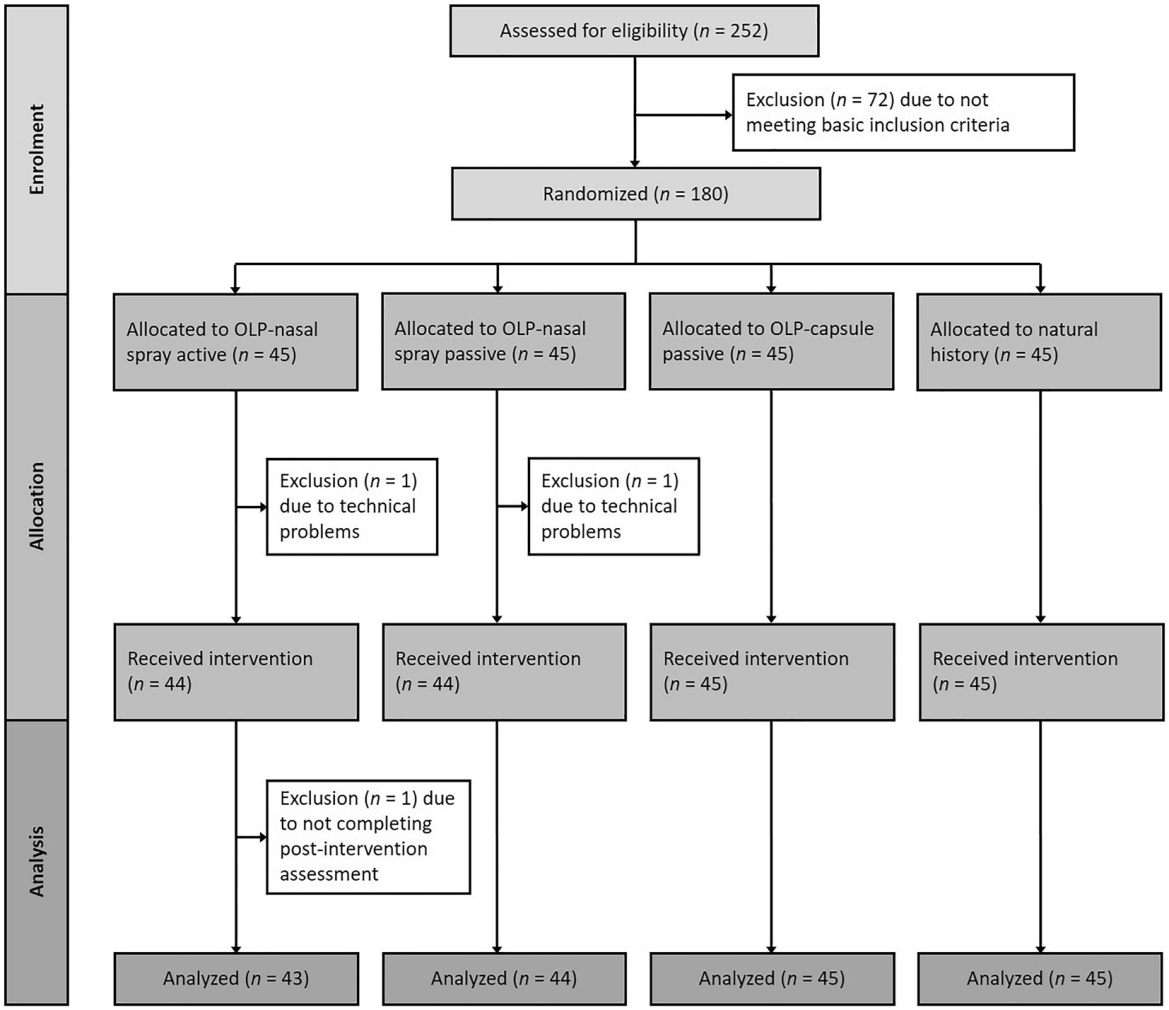
Participant flow chart.

One hundred and eighty highly stressed students (81.5% females) aged between 18 and 34 years (*M* = 23.70, *SD* = 3.13) were recruited at a German university via an e-mail openly advertising a study about an open-label placebo treatment for stress, depressive symptoms, psychological distress and sleeping difficulties. The following inclusion criteria were assessed via a phone interview: (a) age between 18 and 69 years; (b) a Perceived Stress Scale-10 (PSS-10^45^) score of 20 or higher; and (c) fluency in German. In this study, we defined highly stressed university students as individuals with a PSS score higher than one standard deviation above the mean of the student subsample (*M* = 13.27, *SD* = 6.52) reported in a PSS validation study in a representative German community sample^45^. Exclusion criteria were (a) allergies to any substance used in this study (sesame oil and capsaicin) and (b) severe asthma. A sample size of *N* = 164 was calculated based on an a priori power analysis (G*Power 3.1.9.2) for the interaction effect of group and time, assuming a small effect size of *f* = 0.15 with a power of 90% (*α* = 0.05, *r* = 0.50). Assuming a dropout rate of about 10%, *N* = 180 were recruited. Three participants were excluded due to technical problems or not completing the post-intervention assessment (for an overview, see Fig. 1). All participants were informed about the study procedure, gave written informed consent and were reimbursed with €25. The experiment was conducted according to the Declaration of Helsinki and approved by the local ethics committee (Die lokale Ethik-Kommission des Fachbereichs 06 der Justus Liebig Universität Giessen; 2020–0024). The study was retrospectively registrated at the German Clinical Trials Register (DRKS) on 01/02/2023 (DRKS-ID: DRKS00030987; https://drks.de/search/de/trial/DRKS00030987). Participants were randomly assigned to the active OLP nasal spray group, the passive OLP nasal spray group, the OLP capsule group or the natural history group. The groups did not differ with regard to age, number of females, number of study semesters, number of stressful life events within the last week and treatment expectation at baseline (see Table 1). The data collection took place during the COVID-19 pandemic (09/2020 to 11/2020).

**Table 1:** Sample characteristics and group differences at baseline.

	OLP nasal active (*n* = 44)	OLP nasal passive (*n* = 44)	OLP capsule (*n* = 45)	Natural history (*n* = 45)	Group differences
Age in years, *M (SD)*	24.55 (3.02)	23.34 (3.10)	23.38 (2.76)	23.53 (3.54)	*F*(3,177) = 1.48, *p* = .222
Number of females, n (%)	33 (75.0%)	37 (84.1%)	38 (84.4%)	37 (82.2%)	χ^2^(6) = 3.27, *p* = .775
Number of study semesters, *M (SD)*	6.91 (4.24)	6.59 (4.37)	6.27 (3.57)	6.36 (3.77)	*F*(3,177) = 0.23, *p* = .876
Number of stressful life events within the last week, *M* (*SD*)	2.00 (1.31)	2.02 (1.39)	1.98 (1.52)	2.31 (1.36)	*F*(3,176) = 0.56, *p* = .640
Treatment expectation, TEX-Q	5.81 (0.99)	5.82 (0.99)	6.00 (1.07)	5.96 (1.08)	*F*(3,177) = 0.38, *p* = .771

*M* mean; *SD* standard deviation*; n* number of participants; *TEX-Q* Treatment Expectation Questionnaire, ranging from 0 to 10.

### Outcome measures

#### Stress and depression (primary outcomes)

Stress was assessed using the *Perceived Stress Scale-10* (PSS-10^45,46^), a widely used instrument for assessing perceived stress within the last month. The time interval was adjusted to the “past week” allowing the effect of the one-week intervention to be specifically captured. It comprises 10 items, measuring how unpredictable, uncontrollable and/or overloaded participants felt in the past week. The items have to be answered on a Likert scale from “never” (0) to “very often” (4), resulting in a score ranging from 0–40. Cronbach’s alpha in the original study was α = 0.78^45^.

Depressive symptoms were assessed using the depressive symptoms subscale of the *Depression Anxiety and Stress Scale 21* (DASS-21^19^), shortened and translated into German^47^. The DASS-21 is a 21-item self-report measure of state negative affect in the past week, developed with the specific aim of differentiating between depressive symptoms, anxiety and tension/stress. Each subscale comprises 7 statements to be answered on a 4-point Likert-type scale (0 = “Did not apply to me at all” —3 “Applied to me very much, or most of the time”), resulting in a sum score of 0–21 for each subscale. The German DASS-21 scale has good convergent and discriminant validity and high internal consistency (Cronbach’s α = 0.91—depressive symptoms subscale, α = 0.82—anxiety subscale, α = 0.89—stress subscale)^47^.

#### Anxiety, somatization, sleep and well-being (secondary outcomes)

Anxiety symptoms were assessed using the anxiety symptoms subscale of the *Depression Anxiety and Stress Scale 21 (DASS-21*; see primary outcomes above).

Somatization symptoms were assessed using the German translation^48^ of the *Patient Health Questionnaire 15* (PHQ-15^49^). The PHQ-15 is a 15-item, self-report measure assessing the severity of various somatic symptoms (e.g., pain, shortness of breath, feeling faint). The time interval was adjusted such that participants rated how much they had been affected by each symptom in the past week (0 = “not bothered at all” to 2 = “bothered a lot”). Sum scores range from 0–30. The German translation of the PHQ-15 has a good internal reliability of Cronbach’s α = 0.79^50^.

Sleep quality was assessed using the German translation^51^ of the *Pittsburgh Sleep Quality Index* (PSQI^52^), which assesses sleep quality for the previous month (e.g., “during the past month, how often have you had trouble sleeping because you wake up in the middle of the night or early morning?”). For the present study, the time interval was adjusted to the past week. Based on the reported frequency of sleep problems, each of the 19 items is weighted on a 0 (positive extreme) to 3 (negative extreme) interval scale. The global PSQI score is calculated by totaling seven sleep components, providing a range from 0 (very good sleep) to 21 (very bad sleep). The PSQI is widely used in insomnia research^53^; the German version also has high reliability and validity with a high internal consistency of Cronbach’s α = 0.85^51^.

Mental well-being was assessed using the German version^54^ of the *Warwick–Edinburgh Mental Wellbeing Scale (WEMWBS*^55^). This consists of 14 positively worded statements covering subjective well-being and psychological functioning within the last two weeks, adapted to a one-week period for our study. The scale is scored by summing up the response to each item answered on a Likert scale from 1 (none of the time) to 5 (all of the time), thus ranging from 14–70. The German version of the WEMWBS has demonstrated high reliability and validity with an internal consistency of α = 0.92^56^.

#### Previous treatment experiences and treatment expectations (exploratory outcomes)

Treatment expectation was assessed with the *Treatment Expectation Questionnaire (TEX-Q*^57,58^), a generic, multidimensional measure to assess patient expectations for medical and psychological treatments on six subscales (treatment benefit, positive impact, adverse events, negative impact, process, behavioral control), for example, “How much relief in your symptoms do you expect from the treatment?” Each of the 15 items is answered on an 11-point Likert scale (0–10) with item-specific anchors, the lower anchor always indicating no expected change. The average item score (mean of all items after inverting negative items, 0–10) was calculated. As recommended by the authors^59^ the general term ‘treatment’ was replaced with the specific treatment under investigation. The TEX-Q has recently been validated^59^ and demonstrated good internal consistency (α = 0.71-0.92) and moderate to high test–retest reliability (*r* = 0.39-0.76).

Prior treatment experiences and perceived improvement were assessed with the *Generic rating scale for previous treatment experiences, treatment expectations, and treatment effects (GEEE*^60^). In addition to patients’ treatment-related expectations (“How much improvement do you expect from the treatment”), the GEEE assesses both prior treatment experiences (“How much improvement have you experienced with the treatment in the past?”) and current experiences of treatment-related effects (“How much improvement have you experienced since your participation in this study?”). Importantly, treatment expectations, as well as prior and current experiences and potential side effects, are assessed using 0–10 Numeric Rating Scales (NRS), which is similar to the Treatment Expectation Questionnaire TEX-Q. The GEEE is currently under further evaluation in a series of different studies.

##### Pre- and post-intervention ratings

Before the OLP intervention, all participants were asked to indicate which OLP form (nasal spray, capsule) or no OLP they considered to be most effective. Upon completion of the post-intervention assessment, participants within the OLP groups were asked to indicate how often they had missed taking the OLP product and whether they believed that the OLP product did not actually contain an active ingredient.

### OLP intervention

**Figure 2:**
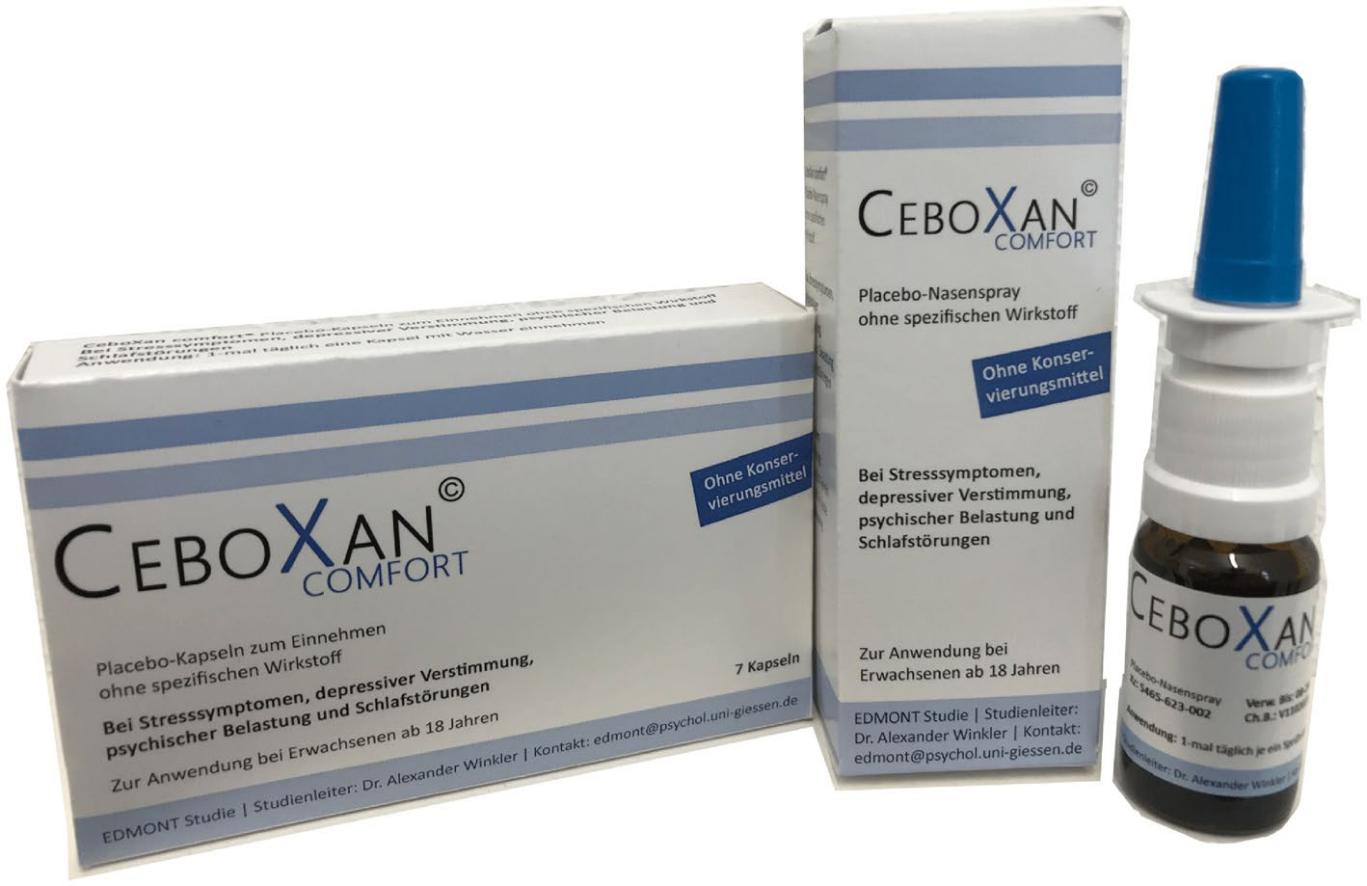
CeboXan Comfort OLP capsules and nasal spray presented as placebo without active ingredient for the treatment of psychological distress.

We developed CeboXan Comfort as the OLP intervention presented as a placebo without an active ingredient for the treatment of psychological distress. CeboXan Comfort was available as capsules and as a nasal spray (see Fig. 2). In order to booster treatment expectation, the package design served as context factor and was modeled after the design of active medication.

#### Placebo capsules and nasal spray

Both the placebo capsules and the placebo nasal spray were delivered in a printed pharmaceutical package, which included a package insert and a blister filled with seven white cellulose capsules or a labeled nasal spray bottle with pump dispenser, respectively. The package provided information on the name of the placebo along with administering information, such as symptom indication and dosage instructions. In the active nasal spray condition only, information on side effects was provided. In addition, similarly to a typical medication, general information (e.g., “keep out of reach of children”, “without preservatives”, “for use in adults 18 years of age or older”, “produced in Germany”, “store in a cool and dry place and away from light”), expiration date, fake charge number and a series of fake barcodes and numbers were displayed on the package. The passive nasal spray contained only sesame oil, so that neither the passive nasal spray nor the placebo capsules produced any noticeable side effects. The active nasal spray contained sesame oil and a small amount of capsaicin (0.0007%) to induce a prickling sensation in the nose in order to mimic side effects and to simulate the onset of a drug-like effect. Participants were told that potentially appearing sensations were induced by the filling material and were assured that there was no active pharmacological ingredient in the nasal spray. Participants of both nasal spray groups received the same information. The experimenter and the participants were blinded to group allocation (active vs. passive nasal spray).

#### OLP rationale

The OLP rationale was given in the same way as in our experimental OLP laboratory study^16^. Participants were first asked about what they know about placebos and the placebo effect. They were given a standardized definition of placebos as being “inert substances without any active pharmaceutical ingredient”. In light of the importance of the provider–patient interaction e.g.,^12^, detailed information about the placebo effect was provided by the experimenter in a 20-min semistructured interactive conversation and complemented by the presentation of two videos (expert lecture and expert statement). Similar to Kaptchuk et al.^61^, the experimenter first explained that “the open label placebo effect is very powerful” (#1) and supported this claim by presenting the results of the Hahn et al.^16^ study on induced sadness as well as a list of several OLP studies demonstrating symptom improvements in various clinical conditions. Next, participants were introduced to the principle of classical conditioning using Pavlov’s dog for illustrative purposes. It was explained that, due to classical conditioning, “the body can automatically respond to taking placebos by activating a self-healing process” (#2). Participants were further told that “positive (explicit) expectations can help but are not necessary” (#3). Finally, it was emphasized that “taking the placebo consistently is important” (#4).

Next, participants were shown two videos that reiterated the delivered information. In the first video, a post-doctoral fellow (AW) gave a lecture about OLPs entailing the same four key points as described above. In the second video, a professor of psychology (CH) outlined the clinical relevance of placebo research. These two experts were introduced as advanced researchers with a high reputation in the field of placebo research. The total duration of these two videos was about 10 min.

Providing the OLP rationale took approximately 30 min. In order to standardize the interaction between experimenter and participant, a semistructured script was used. The experimenters comprised three female psychology students, each wearing a white laboratory coat. The experimenters were instructed to be supportive and confident. Experimenters were also instructed on how to answer questions and to openly address any doubts the participants may have. To minimize any experimenter effects, all three experimenters were trained extensively, practiced the experimental procedure several times under supervision and provided feedback to each other.

### Experimental setup

A randomized parallel group experimental design was used with participants randomly assigned to one of the four groups (active OLP nasal spray, passive OLP nasal spray, passive OLP capsules, natural history). Block randomization was conducted by the principal investigator prior to the laboratory session to allocate participants equally to all groups using a random number generator. Experimenters, who enrolled and assigned participants were not blind to group allocation (nasal spray vs. capsule vs. natural history) but blinded to active vs. passive nasal spray.

**Figure 3:**
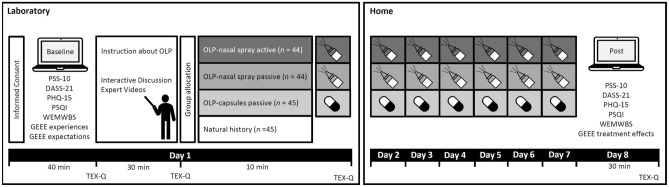
Experimental design.

After giving informed consent, participants were asked to fill out the baseline measures on a PC in the laboratory. Next, all participants received the same highly standardized information about the OLP rationale (see OLP rationale above). Participants were then informed about their group allocation and participants in each of three OLP groups received the OLP capsules or nasal spray, accordingly. The investigator handed over the nasal spray or the capsules (wearing rubber gloves), gave exact instructions on how to use the spray or the capsules and supervised the intake of the first of the seven doses. Participants in the OLP groups were then instructed to take the OLP capsules or nasal spray at home on the following six consecutive days. To enhance adherence, participants were also asked to fill out a paper and pencil protocol (including the day and time of OLP intake). Participants in the natural history group underwent the same procedure without receiving placebo treatment. After 7 days, all participants were invited via email to fill out the post-intervention measures online on study day 8, i.e., one day after the last OLP intake (see Fig. 3). The TEX-Q was assessed at four different assessment points (see Fig. 3). At the third and fourth assessment point the TEX-Q was assessed exclusively in the OLP groups. At the last assessment point, treatment expectation for potential future OLP treatments was assessed.

### Study procedure

Eligibility was determined in a semistructured telephone interview with interested individuals prior to being invited to the lab. Since the study was conducted during the COVID-19 pandemic, the participants were called on the eve of the laboratory appointment to verify that they were not experiencing symptoms of COVID-19 infection. The experimenter wore a white lab coat and an FFP-2 mask during the whole lab session and followed further hygiene regulations such as 1.5 m distance and extensive room disinfection. Participants were tested in a lab with the experimenter in the same room, so that the participants could communicate with the experimenter at any time. The laboratory session followed a standardized protocol. After giving informed consent, participants were asked to complete the questionnaires during the lab session using an online survey platform (Unipark; Questback, 2018); they received information about OLP and were randomly assigned to one of four groups, as shown in Fig. 3. After the lab appointment, all participants were asked to fill out the post-intervention measures online on study day 8. The study lasted about 110 min in total.

### Statistical analysis

Statistical analyses were performed with IBM SPSS Statistics 28.0 for Windows (IBM, Armonk, NY/USA). Significance level was set at α = 0.05 and tests were two-tailed. Differences in baseline sample characteristics, prior treatment experiences, treatment expectation at baseline and perceived improvement post-intervention were analyzed using univariate analysis of variance (ANOVA) and χ^2^-tests. To assess group differences in primary and secondary outcomes, a 4 × 2 mixed ANOVA with group as between-factor (active OLP nasal spray, passive OLP nasal spray, passive OLP capsules, natural history) and time as within-factor (pre- vs. post- OLP treatment) was conducted. A significant group x time interaction in the ANOVA was followed up by planned contrasts comparing all OLP interventions combined and natural history (contrast 1), OLP nasal spray (active and passive) versus OLP capsule (contrast 2) and active OLP versus passive OLP interventions (passive nasal spray and OLP capsule; contrast 3). Since only one participant did not complete the post-treatment assessment, an intent-to-treat analysis was not considered necessary. Additionally, the Reliable Change Index (RCI) as a measure of reliable change was calculated for all primary outcomes and compared between groups via χ^2^-tests. Cronbach’s alpha of the PSS-10 (α = 0.84) and the depressive symptoms scale of the DASS-21 (α = 0.91) was used as reliability coefficient to calculate RCI. Change of treatment expectation in the OLP groups was analyzed using a 3 × 4 mixed ANOVA with group (active OLP nasal spray, passive OLP nasal spray, OLP capsule) as between-factor and time as within-factor (pre-OLP rationale, post-OLP rationale, after the first OLP intake, after the last OLP intake). Simple main effects followed a significant main effect of time.

## Results

Before OLP treatment started, 74.2% of participants, regardless of their group assignment, reported that they expected CeboXan capsule to have the highest effectiveness as compared to 23.6% who indicated the nasal spray and 2.2% who endorsed no OLP treatment as being the most effective. Treatment compliance of the participants in the OLP groups was good. Only 4.5% of the participants reported that they had missed taking CeboXan Comfort on one day. After the OLP treatment, 98.5% of the participants in the OLP groups were convinced that CeboXan Comfort had no active substance, indicating a high credibility of the treatment, while 1.5% believed they had been deceived about the ingredients of CeboXan Comfort.

### OLP effect on stress and depressive symptoms as primary outcomes

With regard to stress symptoms, the 4 × 2 ANOVA yielded only a trend towards a significant interaction effect group x time (*F*[3, 173] = 2.22, *p* = 0.088, partial *η*^2^ = 0.037, see Table 2). Overall, stress symptoms decreased from pre- to post-intervention (main effect time: *F*[1, 173] = 99.64, *p* < 0.001, partial *η*^2^ = 0.365). Also, groups differed significantly (*F*[3, 173] = 2.91, *p* = 0.036, partial *η*^2^ = 0.048). In terms of depressive symptoms, the 4 × 2 ANOVA yielded a significant interaction effect group x time (*F*[3, 173] = 3.97, *p* = 0.009, partial *η*^2^ = 0.064, see Table 2). Moreover, there was a significant reduction in the depressive symptoms (main effect time: *F*[1, 173] = 46.36, *p* < 0.001, partial *η*^2^ = 0.211). There was no significant main effect of group (*F*[3, 173] = 1.25, *p* = 0.294, partial *η*^2^ = 0.021).

**Table 2:** Primary and secondary outcomes at baseline and post-OLP intervention per group, main effects (group, time) and group x time interaction.

	Active OLP nasal spray (*n* = 43)				Passive OLP nasal spray (*n* = 44)				OLP capsule (*n* = 45)				Natural history (*n* = 45)				Main effect group		Main effect time		Group x time interaction	
	Baseline assessment		Post-OLP assessment		Baseline assessment		Post-OLP assessment		Baseline assessment		Post-OLP assessment		Baseline assessment		Post-OLP assessment							
	*M*	*SD*	*M*	*SD*	*M*	*SD*	*M*	*SD*	*M*	*SD*	*M*	*SD*	*M*	*SD*	*M*	*SD*	*F (3,173)*	*p*	*F (1,173)*	*p*	*F (3,173)*	*p*
Primary outcomes																						
Stress (PSS-10)	23.33	6.03	16.74	6.05	25.00	5.19	20.32	6.90	23.62	5.33	19.93	6.39	24.56	4.44	20.98	5.59	2.91	.036	99.64	< .001	2.22	.088
Depression (DASS-21)	7.21	5.72	3.74	4.33	8.45	5.15	6.14	4.67	6.71	4.86	5.38	5.12	6.73	4.30	5.91	4.89	1.25	.294	46.36	< .001	3.97	.009
Secondary outcomes																						
Anxiety (DASS-21)	4.51	3.57	2.16	2.94	6.14	3.96	3.80	4.04	5.16	3.80	3.40	4.22	5.33	3.67	4.07	3.57	1.89	.134	72.72	< .001	1.33	.266
Somatization (PHQ-15)	8.40	4.11	5.58	3.76	10.55	4.71	7.25	4.38	8.89	4.14	7.36	4.30	9.56	4.63	8.22	4.03	2.51	.061	53.88	< .001	2.47	.063
Sleep Quality (PSQI)	7.69	2.98	6.29	3.14	7.93	3.20	6.83	2.71	7.60	2.87	6.42	2.82	7.98	3.61	7.35	2.90	0.60	.618	29.34	< .001	0.67	.571
Well-being, (WEMWBS)	43.35	8.06	47.26	9.58	38.98	8.12	43.02	9.54	42.33	7.18	45.00	8.77	41.24	7.48	42.49	8.74	2.75	.044	29.08	< .001	1.41	.241

*M* mean; *SD* standard deviation; *n* number of participants; *PSS-10* Perceived Stress Scale (ranging from 0 to 40); *DASS-21* Depression Anxiety and Stress Scale (each scale ranging from 0 to 21); *PHQ-15* Somatization scale from the Patient Health Questionnaire (ranging from 0 to 30); *PSQI* Pittsburgh Sleep Quality Index (ranging from 0 to 21); *WEMWBS* Warwick–Edinburgh Mental Wellbeing Scale (ranging from 14 to 70).

**Figure 4:**
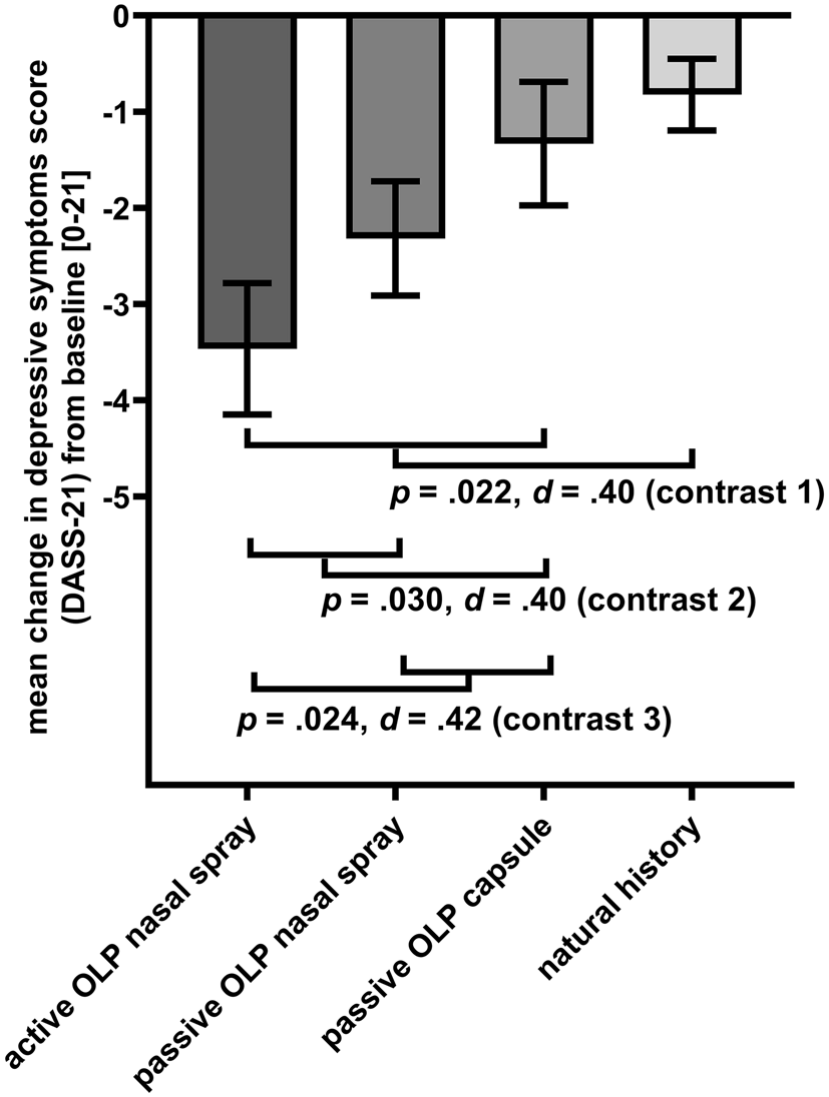
Planned contrasts for change in depressive symptoms (*M*, *SE*; DASS-21) from pre- to post-intervention for all groups (contrast 1: all OLPs vs. natural history; contrast 2: OLP nasal spray (passive + active) vs. capsule; contrast 3: active OLP vs. passive OLP (nasal spray, capsule)).

Planned contrasts comparing all OLP interventions combined with the natural history group (contrast 1) yielded a significant difference in depressive symptoms change scores (∆_post_ _−_ _pre intervention_ =  − 1.55, *SE* = 0.67, *p* = 0.022, *d* = 0.40; see Fig. 4). Depressive symptoms decreased significantly more in the OLP nasal spray groups (active + passive) versus the OLP capsule group (contrast 2: ∆_post_ _−_ _pre intervention_ = 1.56, *SE* = 0.71, *p* = 0.030, *d* = 0.40). Finally, the active OLP group showed significantly greater improvement than the passive nasal spray and capsule OLP interventions (contrast 3: ∆_post_ _−_ _pre intervention_ =  − 1.64, *SE* = 0.72, *p* = 0.024, *d* = 0.42).

**Figure 5:**
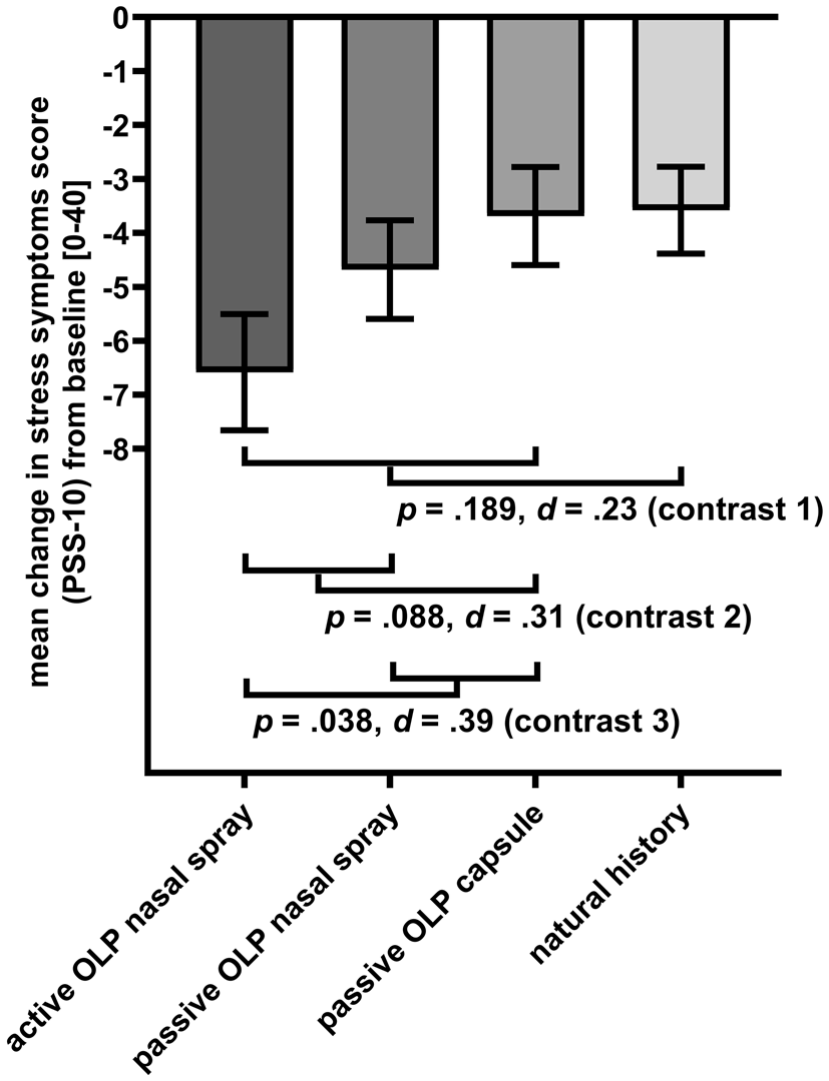
Planned contrasts for change in stress symptoms (*M*, *SE*; PSS-10) from pre- to post-intervention for all groups (contrast 1: all OLPs vs. natural history; contrast 2: OLP nasal spray (passive + active) vs. capsule; contrast 3: active OLP vs. passive OLP (nasal spray, capsule)).

Planned contrasts comparing any OLP intervention and natural history (contrast 1) yielded a nonsignificant difference in stress change scores (∆_post_ _−_ _pre intervention_ =  − 1.41, *SE* = 1.07, *p* = 0.189, *d* = 0.23; see Fig. 5). There was a nonsignificant difference in stress change scores between groups receiving the OLP nasal spray versus OLP capsules (contrast 2: ∆_post − pre intervention_ = 1.94, *SE* = 1.13, *p* = 0.088, *d* = 0.31). There was a statistically significant difference in stress change scores between active OLP versus passive OLP interventions (contrast 3: ∆_post − pre-intervention_ =  − 2.40, *SE* = 1.15, *p* = 0.038, *d* = 0.39) that has to be interpreted with caution due to the trend towards the significant time x group interaction of the 4 × 2 ANOVA reported above.

#### Reliable change

The proportion of participants within each group with a Reliable Change Index (RCI) > 1.96 is summarized in Table 3. There was no significant difference between groups (χ^2^(3) = 3.41, *p* = 0.332, φ = 0.14) with regard to the number of participants with a reliable reduction in stress symptoms (natural history: 35.6%, OLP capsule: 40.0%, passive OLP nasal spray: 47.7%. active OLP nasal spray: 53.5%). Compared to the natural history group (8.9%), there were over three times more participants with a reliable change in depressive symptoms in the active OLP nasal spray group (32.6%), more than twice as many in the passive OLP nasal spray group (27.3%) and twice as many (22.2%) in the OLP capsule group (*χ*^2^(3) = 7.83*, p* = 0.050*, φ* = 0.21).

**Table 3:** Number of participants with Reliable Change Index (RCI) > 1.96 as measure of reliable change in stress symptoms and depressive symptoms per group.

	Active OLP nasal spray (*n* = 43)		Passive OLP nasal spray (*n* = 44)		OLP capsule (*n* = 45)		Natural history (*n* = 45)	
	*n*	*%*	*n*	*%*	*n*	*%*	*n*	*%*
Stress symptoms, PSS-10	23	53.5	21	47.7	18	40.0	16	35.6
Depressive symptoms, DASS-21	14	32.6	12	27.3	10	22.2	4	8.9

*n* number of participants; *PSS-10* Perceived Stress Scale; *DASS-21* Depression Anxiety and Stress Scale.

### OLP effect on anxiety, somatization symptoms, sleep quality and mental well-being

The 4 × 2 ANOVA yielded no significant interaction of group x time for anxiety symptoms (*F*[3, 173] = 1.33, *p* = 0.266, partial *η*^2^ = 0.023), somatization symptoms (*F*[3, 173] = 2.47, *p* = 0.063, partial *η*^2^ = 0.041), sleep quality (*F*[3, 173] = 0.67, *p* = 0.571, partial *η*^2^ = 0.012) or mental well-being (*F*[3, 173] = 1.41, *p* = 0.241, partial *η*^2^ = 0.024), as displayed in Table 2. There was a significant reduction of symptoms (main effect time) for anxiety symptoms (*F*[1, 173] = 72.72, *p* < 0.001, partial *η*^2^ = 0.296), somatization symptoms (*F*[1, 173] = 53.88, *p* < 0.001, partial *η*^2^ = 0.237), sleep problems (*F*[1, 173] = 29.34, *p* < 0.001, partial *η*^2^ = 0.149) and improvement of mental well-being (*F*[1, 173] = 29.08, *p* < 0.001, partial *η*^2^ = 0.144) over time, as displayed in Table 2. Moreover, there were no significant main effects of group for anxiety symptoms (*F*[3, 173] = 1.89, *p* = 0.134, partial *η*^2^ = 0.032), somatization symptoms (*F*[3, 173] = 2.51, *p* = 0.061, partial *η*^2^ = 0.042) or sleep quality (*F*[3, 173] = 0.60, *p* = 0.618, partial *η*^2^ = 0.011), except for mental well-being (*F*[3, 173] = 2.75, *p* = 0.044, partial *η*^2^ = 0.046), as displayed in Table 2.

### Prior treatment experiences, treatment expectations, perceived improvement

At baseline, there were no significant group differences in prior treatment experiences (*F*[3, 75] = 0.59, *p* = 0.627) or treatment-related expectations (*F*[3, 177] = 0.13, *p* = 0.945). Moreover, after treatment, groups did not differ with regard to perceived improvement (*F*[2, 131] = 0.11, *p* = 0.896).

**Figure 6:**
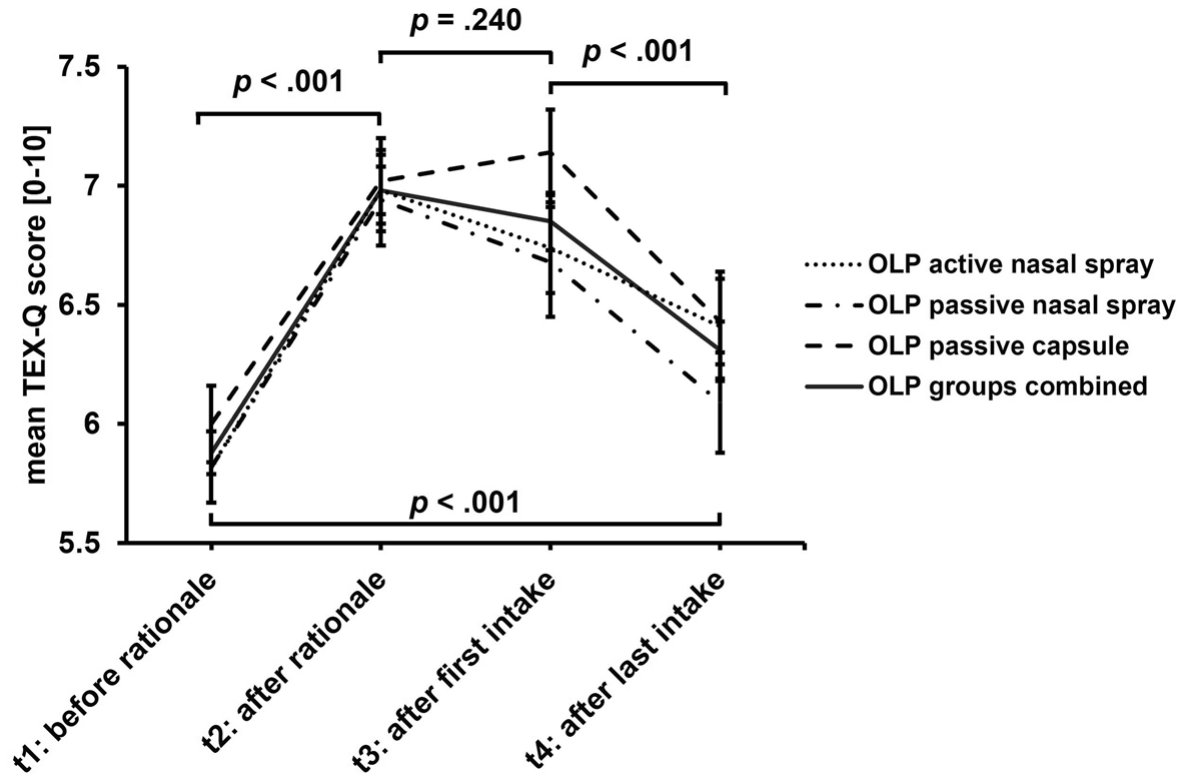
Course of treatment expectation (mean TEX-Q score) across OLP groups.

Treatment expectation (TEX-Q), assessed in the OLP groups, changed significantly over the course of the intervention (*F*[3, 387] = 64.40, *p* < 0.001, partial *η*^2^ = 0.333). Neither the interaction group x time (*F*[6, 387] = 1.14, *p* = 0.338, partial *η*^2^ = 0.017) nor the main effect for group (*F*[2, 129] = 0.70, *p* = 0.504, partial *η*^2^ = 0.011) reached significance. Explaining the OLP rationale led to a significant increase in explicit treatment expectation compared to baseline (∆_t2 − t1_ = 1.10, *SE* = 0.07, *p* < 0.001, *d* = 0.99) across the OLP groups (see Fig. 6). However, the first intake of the OLP directly after the rationale did not further strengthen treatment expectation significantly (∆_t3 − t2_ =  − 0.13, *SE* = 0.06, *p* = 0.240, *d* =  − 0.10). At the end of the intervention, treatment expectation was significantly lower compared to the first intake (∆_t4 − t3_ =  − 0.54, *SE* = 0.10, *p* < 0.001, *d* =  − 0.40), but still significantly higher than at baseline (∆_t4 − t1_ = 0.43, *SE* = 0.11, *p* < 0.001, *d* = 0.36).

## Discussion

Our findings indicate that OLP treatments in highly stressed students had a substantial and rather specific effect on reported depressive symptoms. Groups differed primarily in the improvement of depressive symptoms, but not other outcomes of psychological distress. Reduction of depressive symptoms was higher for participants receiving an OLP nasal spray than for participants receiving an OLP capsule (*d* = 0.40), even though, a priori, OLP capsules were believed to be most effective. Participants receiving an active OLP, thus simulating side effects, benefitted significantly more than participants receiving a passive OLP (*d* = 0.42). Compared to the natural history group, there were more than three times more participants with a reliable change in depressive symptoms in the active OLP nasal spray group, more than twice as many in the passive OLP nasal spray group and twice as many in the OLP capsule group.

We demonstrated for the first time that the pharmaceutical form of an OLP treatment and the simulation of side effects play a substantial role in accounting for OLP effects. Indeed, the obtained OLP effect was larger for the nasal spray compared to the capsules. Studies focusing on the physical characteristics of the placebo capsules suggest that their effectiveness depends on features such as color and shape^62^, price^63^ or brand^64^. Yet, placebo capsules have not yet been systematically compared to other application forms. It is likely that participants had more experiences with pills or capsules for a wide variety of symptoms and with mixed effectiveness. By contrast, a nasal spray is associated primarily with alleviating a stuffed nose, often resulting in rather immediate symptom relief. Based on notions such as embodied cognition, this might activate higher implicit expectations for improvement. Moreover, a nasal spray by its very nature induces immediate sensations in the nose, even when without an active ingredient. Hence, a nasal spray compared to a capsule may be more salient and less fraught with previous treatment experiences, thus eliciting more unconscious positive treatment expectations. Intriguingly, the observed greater benefit for an OLP nasal spray is somewhat at odds with what participants believed. When asked before treatment, most participants across all groups predicted that capsules would have the strongest effect. Clearly, this underlines the need to address unconsciously mediated contextual factors.

We found that an active placebo had a more pronounced effect on depressive symptoms compared to passive placebos, thus complementing similar observations for *deceptive* placebos^44,65^. Given that sensory features and bodily sensations could be especially important in the induction of OLP effects^28,33^, active OLPs may be more effective by possibly shifting attention to bodily sensations and increasing the salience of the OLP treatment. Indeed, nasal sprays produce a more direct, perceivable effect whereas the effect of capsules takes more time. According to predictive coding, this might be especially important when taking an OLP, assuming that top-down predictions are simultaneously compared to sensory information. Perhaps simulated side effects shifts the correction of the prediction error more towards adjusting the internal prediction by shifting it towards expecting less symptoms or by decreasing the precision of the prediction, i.e. increasing uncertainty about what symptoms to expect. The bottom line is, that OLPs differ in the degree of perceivable onset effects depending on their pharmaceutical form and simulated side effects, i.e. an immediate prickling in the nose from the active nasal spray is more salient than the moisturizing effect in the nose from the passive nasal spray, which might be still more salient than swallowing a capsule.

Importantly, the observed beneficial effect of an OLP on depressive symptoms in stressed students extends previous OLP intervention studies. Using a rather comparable design, El Brihi et al.^18^ found a large effect (*ƞ*^2^_p_ = 0.14) of a 7-day OLP intervention on emotional distress in an unselected student sample. As a result of relying on the composite DASS-21-score, symptom specificity of this beneficial effect is unclear. Kleine-Borgmann et al.^17^ reported a smaller increase in stress (*d* = 0.32) for their sample of medical students preparing their mid-annual exams when taking an OLP. It should be noted that participants underwent the OLP intervention primarily to improve cognitive functioning and possibly to improve stress and emotional distress. It is possible that, receiving the intervention during a highly stressful pre-exam period, participants might have particularly focused on perceived stress. While the OLP interventions in the present study yielded a robust (and reliable) reduction in depressive symptoms, subjective stress (assessed with the PSS-10) as well as somatic symptoms showed only a trend to decrease. This rather specific effect on depressive symptoms might be accounted for by our rationale, which strongly focused on sadness-related effects. For example, we presented findings from our previous study^16^ demonstrating a sadness-protective effect. The OLP rationale of El Brihi et al.^18^ did entail the same four discussion points, but these were not described in detail. Possibly, the depressive symptoms scale of the DASS-21 might have the highest sensitivity to change compared to the other outcomes of psychological distress.

We also explored the role of treatment expectation. Interestingly, groups did not differ in their explicit treatment expectation. Following the OLP rationale, explicit treatment expectation increased compared to baseline. However, the first intake of the OLP directly after the rationale did not further strengthen treatment expectation. Over time, treatment expectation decreased significantly, but was still significantly higher than at baseline. Since there were no significant differences between the different pharmaceutical forms, the difference in the efficacy of the OLP groups cannot be accounted for by explicit treatment expectations. Since symptom improvement was most pronounced in the active OLP nasal spray group compared to the other OLP groups, other mechanisms, such as the implicit expectations, possibly instilled or augmented by the sensory features of the intake, may be more important in inducing OLP effects.

Some limitations should be noted. First, our sample of highly stressed university students is rather selective concerning gender, educational level and age. While this limits generalizability, it allows direct comparisons to those of the other OLP studies on psychological distress. Moreover, we included an at-risk sample and determined reliable change, thus allowing clinical implications. The selection of a highly stressed population might induced regression to the mean effects that might overshadowed treatment effects of the OLP conditions. To address this methodological bias, we used a randomized controlled parallel group design including a natural history group. By comparing change in OLP conditions against change in the natural history group, we controlled for regression to the mean effects. Second, the rather specific effect on depressive symptoms may be due to the rationale primarily addressing protection against sadness. Nonetheless, it should be noted that the observed amelioration of perceived stress closely matches the pattern of change in depressive symptoms. Third, we assessed treatment outcomes directly after a relatively short treatment duration of seven days without a follow-up assessment. Fourth, we expected the passive nasal spray to be more effective due to its salience, its being tangible and distinct sensory features compared to the passive oral capsule. In addition, nasal sprays are likely to be strongly associated with relief of (cold) symptom. Nevertheless, oral medication possibly be associated with more invasive treatments than nasal spray and with possible use of anti-depressants. However, this would be primarily expected for participants who have taken antidepressants in the past, but not for the majority of our sample. Finally, we did not assess participants’ coping strategies (e.g., sport, relaxation, social contacts), which might have influenced depressive symptoms. Possibly, the OLP intake might have led participants to engage in adaptive coping.

Future studies should deliberately synchronize the used rationale and the primary outcome, since the rather specific effect of OLPs on depressive symptoms demonstrated in our study, together with the rather small effects in the study by Schienle et al.^26^, emphasize the importance of the alignment of the rationale and the outcome. Our results provide further evidence that, in OLP treatment, explicit treatment expectations seem to play a minor role, while contextual effects such as the pharmaceutical form and simulation of side effects strongly contribute to the OLP effect, possibly by less conscious mechanisms which need to be delineated in more detail. Since we were able to demonstrate a substantial reduction of depressive symptoms through OLP treatment in a sample of stressed students at risk of depression, a promising next step for future studies might be to demonstrate the maintenance of this effect in at risk populations.

In summary, our findings demonstrate that an OLP treatment is efficacious in students with high levels of stress and emotional distress. Most importantly, the observed pattern of improvement suggests that OLP effects are strongly determined by outcome expectations inherently communicated by the OLP rationale, while explicit treatment expectation seems to play a minor role. Consistent with the notion of implicit expectations possibly shaped by previous treatment experiences and activated by salient sensory cues, pharmaceutical form and the simulation of side effects strongly determined OLP efficacy. These findings add to the understanding of mechanisms underlying the open label placebo effect, and provide important insight on how open label placebo effects may be utilized in the prevention and treatment of depression.

## Data Availability

De-identified data for analyses along with a codebook and the data analysis scripts, as well as instructions for participants in German language are publicly posted at zenodo open-access repository (https://doi.org/10.5281/zenodo.7463418). The videos used to induce placebo effects will be provided on demand by the authors.
